# Gait training with partial body weight support during overground walking for individuals with chronic stroke: a pilot study

**DOI:** 10.1186/1743-0003-8-48

**Published:** 2011-08-24

**Authors:** Catarina O Sousa, José A Barela, Christiane L Prado-Medeiros, Tania F Salvini, Ana MF Barela

**Affiliations:** 1 Department of Physical Therapy, Federal University of São Carlos, São Carlos, SP, Rodovia Washington Luis, Km 235, CP, 676, 13656-905, São Carlos, SP, Brazil; 2 Department of Physical Education. São Paulo State University, Rio Claro, SP, Av. 24-A, 1515, Bela Vista, 13506-900, Rio Claro, SP, Brazil; 3 Graduate Program in Human Movement Sciences, Institute of Physical Activity and Sport Sciences, Cruzeiro do Sul University, São Paulo, SP, Rua Galvão Bueno, 868, 13° andar, Bloco B, 01506-000, São Paulo, SP, Brazil

## Abstract

**Background:**

It is not yet established if the use of body weight support (BWS) systems for gait training is effective *per se *or if it is the combination of BWS and treadmill that improves the locomotion of individuals with gait impairment. This study investigated the effects of gait training on ground level with partial BWS in individuals with stroke during overground walking with no BWS.

**Methods:**

Twelve individuals with chronic stroke (53.17 ± 7.52 years old) participated of a gait training program with BWS during overground walking, and were evaluated before and after the gait training period. In both evaluations, individuals were videotaped walking at a self-selected comfortable speed with no BWS. Measurements were obtained for mean walking speed, step length, stride length and speed, toe-clearance, durations of total double stance and single-limb support, and minimum and maximum foot, shank, thigh, and trunk segmental angles.

**Results:**

After gait training, individuals walked faster, with symmetrical steps, longer and faster strides, and increased toe-clearance. Also, they displayed increased rotation of foot, shank, thigh, and trunk segmental angles on both sides of the body. However, the duration of single-limb support remained asymmetrical between each side of the body after gait training.

**Conclusions:**

Gait training individuals with chronic stroke with BWS during overground walking improved walking in terms of temporal-spatial parameters and segmental angles. This training strategy might be adopted as a safe, specific and promising strategy for gait rehabilitation after stroke.

## Background

Typically, individuals with stroke walk slower than their peers and present asymmetry in spatial-temporal parameters [1,2] and joint angles [3]. These typical characteristics may influence the return of pre-stroke conditions [4], mainly because there exists an increased risk of falling [5], followed by decreases in autonomy, and consequently, an increase in social isolation [6,7]. Therefore, reestablishing independence via walking is a crucial goal of any rehabilitation program for individuals with stroke [3,4,8].

Among the different strategies of gait training for individuals with stroke, the use of a partial body weight support (BWS) system has continued to gain popularity [9-13]. This strategy of gait training originated from experiments on animals with complete spinal cord transections [14,15], which established that training on a treadmill promotes the generation of an automatic locomotor pattern by spinal neurons [16,17], named the central pattern generator. Gait training humans affected by stroke using a BWS system on a treadmill increased walking speed and endurance when compared to conventional gait training overground [9] or when using only a treadmill [10].

A BWS system alleviates the body weight of the lower limbs symmetrically [10,18,19], promotes stabilization of the trunk [20], improves balance control, and avoids falls [16]. Most studies had adopted 30% of a subject's body weight unloading due to this percentage's effectiveness on gait training [9,12,21,22]. Additionally, the type of training surfaces used by patients is crucial, and this consideration may facilitate skill transfer to daily life activities [23,24]. To our knowledge, no one has evaluated the effects of gait training with partial BWS during overground walking on the walking performance of individuals with stroke. Previous studies concerning BWS during overground walking investigated changes in gait patterns but not its training effects [22,25-27]. Therefore, the purpose of this study was to investigate the effects of gait training on ground level with partial BWS on temporal-spatial parameters and on lower limb and trunk segmental angles of individuals with chronic stroke during overground walking without BWS. It was hypothesized that these individuals' gait performance would improve after six weeks of the proposed gait training and they would experience reduced asymmetry.

## Methods

### Participants

Twenty individuals with chronic stroke discharged from a conventional rehabilitation program at a physical therapy clinic at the university where this study took place volunteered for this study. After an initial evaluation, which occurred one week before the initiation of gait training and consisted of personal data registration (name, home address, telephone, birth date, time of stroke, type of lesion, reported neurological and orthopedic diseases) and a physical examination (body mass, stature, blood pressure, cardiac and respiratory frequency, paretic body side, level of spasticity, body deformities, functional gait capacity), sixteen individuals were eligible to participate in this study, according to the inclusion and exclusion criteria described in the following paragraph. However, four of these individuals did not complete the gait training program due to previous orthopedic complications (n = 3), not reported on the time of the initial evaluation, or desistance (n = 1). General information of the remaining twelve individuals that completed all the stages of the study is presented on Table 1.

**Table 1:** General information of the participants that completed all the stages of the study

Participant	Gender	Age (years)	Mass (kg)	Height (cm)	Type of Lesion	Hemiparesis	Time of post-stroke (years)
1	M	43	69.8	172	Ischemic	Right	8
2	M	64	72	177	Hemorrhagic	Left	7
3	F	43	76.7	165	Ischemic	Right	1
4	M	50	78.6	176	Ischemic	Right	7
5	F	44	110	169	Ischemic	Right	5
6	M	59	80.8	183	Ischemic	Right	6
7	M	49	97.1	169	Ischemic	Right	1
8	F	56	102.4	163	Ischemic	Left	1
9	M	62	82.3	167	Ischemic	Left	4
10	F	52	66.4	161	Hemorrhagic	Left	6
11	M	55	102	175	Ischemic	Left	1
12	M	61	91.4	163	Ischemic	Left	10

**Mean**	**-**	**53.2**	**85.8**	**170**	**-**	**-**	**4.6**
**SD**	**-**	**7.5**	**14.4**	**6.7**	**-**	**-**	**3.0**

Note: M = male; F = female

Inclusion criteria were: an elapsed time longer than one year since stroke and the ability to walk approximately 10 m with or without assistance. Participants were excluded if: they presented any clinical signs of heart failure (New York Heart Association), arrhythmia, or angina pectoris; orthopedic (n = 2) or other neurological diseases (n = 2) that compromised gait; or severe cognitive or communication impairments. All individuals signed an informed consent agreement approved by the University ethics committee prior to participating in this study in accordance with the Declaration of Helsinki.

### Training sessions

**Figure 1:**
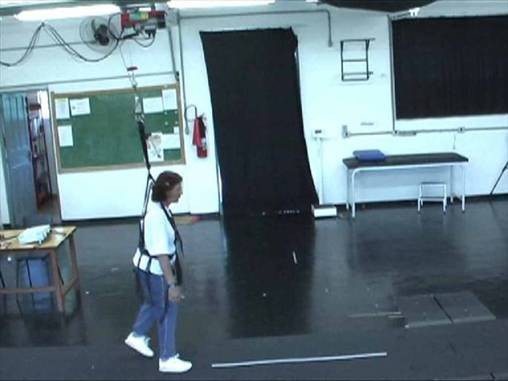
**Partial view of a gait training session with the body weight support system used in the study**. The rail that the electric motor slides along, the load cell, and one of the participants of the study wearing the harness are shown.

Individuals were supported by a horizontal bar equipped with a harness with adjustable straps for the hips and thighs [27], as they walked overground along a 10 m walkway (Figure 1). A steel cable from an electric winch adjusted the horizontal bar vertically and a load cell, connecting the horizontal bar to the cable, measured the amount of weight borne by the BWS system, which was shown on a digital display. Before walking, participants remained still when the winch was activated by the experimenter until adjusting 30% of body weight unloading. After the first three weeks, body weight unloading was adjusted to 20%. Individuals' body mass was measured weekly to ensure that the appropriate percentage of body weight was unloaded.

During training sessions, verbal cues that could improve walking speed and joint excursion were given. Heart rate and blood pressure were observed at the beginning and end of each session and when individuals reported any discomfort during the training session to ensure their safety. Rest periods were allowed during each training session according to individual needs.

All individuals were submitted to 45-minute gait training sessions wearing their walking shoes, three times a week, alternating days during six weeks. No participant was given any other type of physical intervention or conventional gait training, stretching, muscle strengthening or endurance exercises while participating in this study.

### Gait assessment

Individuals were assessed at least one day before the first gait training session and at least one day after the last gait training session (but no longer than one week either before or after the gait training period), walking freely at self-selected comfortable speeds along a 10 m walkway six times. They were videotaped by four digital cameras (AG-DVC7P, Panasonic) at 60 Hz, which were positioned bilaterally allowing simultaneous kinematics measurements of paretic and nonparetic limbs in either direction of motion (from left to right and vice-versa). During the evaluation, individuals were not allowed to use any assistive devices, and when necessary, they walked while holding the index finger of one of the physical therapists to assist their balance, without providing any meaningful mechanical support.

Passive reflective markers were placed on the nonparetic and paretic sides of the body at the following anatomical locations: head of the fifth metatarsal, lateral malleolus, lateral epicondyle of the femur, greater trochanter, and acromion, in order to define the foot, shank, thigh, and trunk segments, respectively. The digitalization and the reconstruction of all markers were performed using the Ariel Performance Analysis System - APAS (Ariel Dynamics, Inc.) software. Filtering and posterior analyses were performed using Matlab software (MathWorks, Inc.). Reconstruction of the real coordinates was performed using the direct linear transformation (DLT) procedure.

### Outcome measures

One intermediate stride (walking cycle) per trial by each individual, for a total of three selected trials, was analyzed. The trial selection was determined by the best visualization of the markers and walking performance in an uninterrupted trial. Through visual inspection, a stride was defined by two consecutive initial contacts of the same limb to the ground along the progression line. Additionally, walking events during a walking stride were identified for subsequent calculation of temporal organization of walking (initial and terminal double stance, single-limb support, and swing period). This procedure was performed for both nonparetic and paretic sides. All data were digitally filtered using a 4^th ^order low-pass and zero-lag Butterworth filter with a cutoff frequency of 8 Hz, defined based upon residual analysis [28].

The following variables were examined: mean walking speed, calculated as the ratio between the distance traveled and its duration (determined by the position of the greater trochanter marker, which is closer to the center of body mass); step length, the distance between initial contact of each foot; stride length, the distance between two successive initial contacts of each foot to the ground (determined by the position of the lateral malleolus marker); stride speed, calculated as the ratio between stride length and duration; duration of total double stance and single-limb support [29], vertical distance between foot and walking surface during swing period - "toe-clearance" (determined by the difference between maximum and minimum vertical position of the marker placed on the fifth metatarsal), and maximum and minimum foot, shank, thigh, and trunk segmental angles during each stride. The conventions adopted to describe segmental rotations were counter-clockwise (backward) and clockwise (forward) rotations around the medial-lateral axis in the sagittal plane, which denoted positive and negative values, respectively [30]. For example, a counter-clockwise rotation of the trunk means trunk extension from the neutral position and a clockwise rotation means trunk flexion from the neutral position.

### Statistical analysis

For all variables, data from three trials under each evaluation were averaged for each participant. A one-way analysis of variance (ANOVA) was conducted, using evaluation (before and after gait training) as a factor and mean walking speed as the dependent variable. Two two-way ANOVAs and six multivariate analyses of variance (MANOVAs) were employed, using body side (nonparetic and paretic) and evaluation as factors. The dependent variables were step length and toe-clearance for the two ANOVAs, and stride length and stride speed for the first MANOVA; durations of total double stance and single-limb support for the second MANOVA; and minimum and maximum foot, shank, thigh, and trunk segmental angles for the third, fourth, fifth, and sixth MANOVAs, respectively. When applicable, univariate analyses and the Tukey post-hoc tests were employed. An alpha level of 0.05 was adopted for all statistical tests, which were performed using SPSS software.

## Results

During evaluations, none of the individuals used assistive devices. However, during the first evaluation (before training) two participants needed assistance from a physical therapist, who offered her index finger, to assist their balance when walking. During the second evaluation (after training) only one participant needed the same type of assistance when walking. All participants expressed interest and motivation throughout the training period, and they disseminated their experiences with the study to other nonparticipants with stroke.

Table 2 depicts the mean and standard deviation (± SD) of gait cycle temporal-spatial parameters before and after gait training for both sides of the body. Individuals walked faster after gait training (F_1,11 _= 8.384, p = 0.015). ANOVA revealed interaction between the sides of the body and evaluation of step length (F_1,11 _= 7.952, p = 0.017). Post-hoc tests indicated that the step length of the nonparetic side was longer than the step length of the paretic side before gait training, and that after gait training step length of the paretic side became similar to the step length of the nonparetic side.

Toe-clearance increased after training (F_1,11 _= 5.609, p = 0.037), and the nonparetic side showed greater toe-clearance than the paretic side, (F_1,11 _= 7.092, p = 0.022). Stride length and speed were also influenced by training (Wilks' Lambda = 0.463, F_1,11 _= 5.789, p = 0.021), with univariate analyses indicating increased stride length (F_1,11 _= 12.040, p = 0.005) and stride speed (F_1,11 _= 7.010, p = 0.023) on both sides of the body after gait training.

Regarding the stance period, MANOVA revealed only a side of the body effect (Wilks' Lambda = 0.085, F_1,11 _= 54.028, p = 0.001). Univariate analysis indicated that the nonparetic side showed a longer single-limb support duration than the paretic side (F_1,11 _= 116.536, p = 0.001).

Table 3 depicts the mean (± SD) of minimum and maximum foot, shank, thigh, and trunk segmental angles during a gait cycle of both sides of the body before and after gait training. MANOVA revealed a training effect (Wilks' Lambda = 0.461, F_1,11 _= 5.856, p = 0.021), and a side of the body effect (Wilks' Lambda = 0.216, F_1,11 _= 18.184, p = 0.001) for minimum and maximum foot segmental angle. Univariate analysis indicated that both counterclockwise (F_1,11 _= 8.187, p = 0.015) and clockwise foot rotation (F_1,11 _= 5.317, p = 0.042) increased after gait training. The nonparetic side presented greater clockwise foot rotation than the paretic side (F_1,11 _= 33.989, p = 0.001).

**Table 2:** Spatial-temporal and toe-clearance data

Outcome measures	Before gait training		After gait training	
	
	Nonparetic	Paretic	Nonparetic	Paretic
Walking speed (m/s)*	0.42 ± 0.23		0.55 ± 0.33	
Step length (m) ***	0.36 ± 0.12**	0.32 ± 0.12**	0.38 ± 0.13	0.40 ± 0.15
Toe-clearance (cm)*	6.19 ± 1.60**	5.01 ± 1.39**	7.35 ± 2.27**	5.49 ± 2.04**
Stride length (m)*	0.65 ± 0.20	0.66 ± 0.20	0.78 ± 0.26	0.79 ± 0.26
Stride speed (m/s)*	0.41 ± 0.22	0.42 ± 0.22	0.53 ± 0.32	0.54 ± 0.32
Double-limb stance (%)	46.38 ± 13.94	46.30 ± 15.32	42.89 ± 16.88	42.64 ± 17.45
Single-limb support (%)	33.48 ± 8.55**	19.25 ± 6.82**	34.20 ± 9.24**	22.10 ± 7.80**

Mean and (± SD) values of outcome measures during stride cycle. Notes: * significant differences (P < 0.05) between evaluations; ** significant differences (P < 0.05) between sides of the body; *** significant interaction (P < 0.05) between the sides of the body and evaluations.

**Table 3:** Minimum (clockwise rotation) and maximum (counter-clockwise rotation) segmental angles

Outcome measures	Before gait training		After gait training	
	
	Nonparetic	Paretic	Nonparetic	Paretic
Foot angle (degrees)				
Minimum*	101.45 ± 8.56**	117.52 ± 15.06**	96.13 ± 13.12**	115.24 ± 12.85**
Maximum*	161.17 ± 5.30	155.76 ± 5.56	163.36 ± 6.83	160.47 ± 6.56
Shank angle (degrees)				
Minimum*	46.32 ± 5.81**	62.80 ± 9.74**	43.62 ± 6.45**	58.88 ± 10.15**
Maximum*	97.97 ± 4.95	96.99 ± 5.46	99.87 ± 5.98	99.09 ± 4.45
Thigh angle (degrees)				
Minimum*	84.03 ± 4.08	85.88 ± 6.98	81.68 ± 5.64	83.15 ± 7.63
Maximum	115.81 ± 2.88	112.95 ± 4.53	116.55 ± 2.80	115.75 ± 3.83
Trunk angle (degrees)				
Minimum	79.89 ± 3.61**	75.58 ± 4.45**	79.79 ± 2.71**	76.00 ± 5.99**
Maximum	88.41 ± 3.96	88.71 ± 4.80	89.11 ± 3.85	90.65 ± 4.69

Mean (± SD) values of outcome measures during stride cycle. Note: * significant differences (P < 0.05) between evaluations; ** significant differences (P < 0.05) between sides of the body

Similarly, MANOVA revealed a training effect (Wilks' Lambda = 0.337, F_1,11 _= 9.822, p = 0.004) and a side of the body effect (Wilks' Lambda = 0.131, F_1,11 _= 33.200, p = 0.001) for minimum and maximum shank segmental angles. Univariate analysis indicated that both counterclockwise (F_1,11 _= 11.669, p = 0.006) and clockwise rotations (F_1,11 _= 10.156, p = 0.009) increased after gait training. The nonparetic side presented greater clockwise shank rotation than the paretic side (F_1,11 _= 56.942, p = 0.001).

MANOVA revealed training effect only for thigh minimum and maximum segmental angles (Wilks' Lambda = 0.435, F_1,11 _= 6.503, p = 0.016) with an increased thigh clockwise rotation (F_1,11 _= 7.544, p = 0.019).

Finally, MANOVA revealed a side of the body effect for trunk minimum and maximum segmental angles (Wilks' Lambda = 0.294, F_1,11 _= 12.029, p = 0.002). Univariate tests indicated lower clockwise trunk rotation on the nonparetic side when compared to the paretic side (F_1,11 _= 11.667, p = 0.006).

## Discussion

This study investigated the effects of gait training on ground level with partial BWS on temporal-spatial parameters and the lower limb and trunk segmental angles of individuals with chronic stroke during overground walking with no BWS. Several aspects of gait in the individuals with stroke were improved, such as increased walking speed, symmetrical steps, longer and faster strides, and increased toe-clearance. Although these individuals increased rotation of foot, shank, thigh, and trunk segmental angles on both sides of the body, they still presented body side asymmetry on foot, shank, and trunk segments, after gait training. Therefore, our hypothesis that six weeks of gait training with BWS during overground walking would improve walking performance of individuals with chronic stroke was partially confirmed with the exception of asymmetry of both sides of the body that remained for foot, shank, and trunk segments. However, step length did become symmetrical.

To our knowledge, this was the first attempt to implement a gait training strategy for individuals with chronic stroke with partial BWS on a level surface and the results were promising. Although this gait training strategy was employed only for six weeks, gait speed and step symmetry indicated that the training protocol promoted motor recovery; these two parameters are important indicators of recovery for individuals with stroke [3,31,32]. Walking on a treadmill leads to symmetrical steps as compared to overground [33]. However, in this study gait training with BWS during overground walking also promoted step symmetry. Improvements were also observed in stride length and speed which may have contributed to increases in walking speed which, in sum, indicates the functional improvement of balance [29], and might provide more autonomy.

Among different measurements, gait speed is the most investigated in clinical gait studies to verify the interventional effects [34]. Gait speed is chosen primarily because the final attained walking speed is essential for ambulation in both indoor and outdoor environments [35,36]. The gait training strategy adopted in the present study was as effective for increasing walking speed as previous studies that submitted individuals with chronic stroke to: isokinetic training for lower extremities [37], home-based exercises [38], treadmill and overground walking without BWS [39], treadmill with BWS [40], and treadmill with BWS combined with overground without BWS [41]. Our results suggest that training with BWS during overground walking effectively increases walking speed of individuals with chronic stroke.

Individuals with stroke present limited foot rotation and lower-limb flexion during the swing period [42], which leads to insufficient toe-clearance. Consequently, these individuals have an increased risk for stumbling and falling [5]. Besides increasing gait speed, gait training with partial BWS during overground walking promoted increased toe-clearance which is an important gait requirement for safety. Increased toe-clearance resulted from increased segmental rotation of the lower limbs. These results may suggest that the training protocol promoted voluntary responses of lower-limb muscles, which then may have generated more strength and power, because the participants in this study presented greater motion and control of foot and shank segments after training. It is important to note that although the use of BWS during overground walking limits hip movement [27], these individuals increased clockwise rotation of the thigh after training.

Aside from these promising improvements, our training protocol did not change the asymmetry of gait cycle temporal organization (duration of single stance) and segmental angles, which is a discriminating factor in individuals with stroke [43]. Harris-Love et al. [18] found that individuals with chronic stroke presented different durations of single stance and stance/swing ratios between paretic and nonparetic limbs even during treadmill walking. The participants of this study did not improve these gait characteristics because they were in a chronic recovery stage, which contributed to a consolidated gait pattern [3] and, was therefore much more difficult to change by the adopted protocol intervention constituted only by 18 sessions of gait training. This pattern may be considered a compensatory strategy that these individuals have adopted to propel the paretic limb forward.

Finally, an important aspect that characterizes our protocol involving gait training with partial BWS during overground walking is the safety which motivated individuals to participate with a high level of adherence. When asked about the training protocol, all individuals answered that they felt safe. Consequently, these individuals experienced their gait improvement as they became more confident in managing deambulation by themselves. Although hard to quantify, individual's safety and confidence are definitive and critical aspects of any intervention protocol.

Although gait training with partial BWS during overground walking protocol was promising, this study had some limitations. First, we adopted 30% of BWS for the first three weeks of gait training because it was the most commonly applied percentage of body weight unloading used during gait training with BWS on treadmill [9,12,21]. BWS was then reduced to 20% during the last three weeks to increase the activation of the lower-limb muscles and energy expenditure [44]. In future studies, initiating gait training with less than 30% of BWS may improve recovery since this percentage seems to difficult force production [27] which is required for forward propulsion. This factor is different, for instance, on a treadmill. More importantly, body unloading should be adjusted individually, without one standardized reduction for everyone. Second, only kinematics analysis in the sagittal plane was investigated; in future studies kinetics and muscle activation should be targeted. Third, we were unable to verify the maintenance of the improved gait performance because these participants enrolled in a different training protocol following this study. Follow up should be employed in future studies, including a measurement of community ambulation as suggested by Lord and Rochester [45], to verify if the benefits of this gait training strategy are preserved. Next, individuals with stroke walking on ground level with BWS were not compared to a control group such as individuals with stroke walking either with BWS on a treadmill or with no BWS. It is important to compare, for example, the two types of surfaces with the same therapist in future studies to quantify the maintenance of interest and motivation throughout the training period and report how this important aspect of the intervention protocol affects results.

## Conclusions

Gait training with BWS during overground walking improved the gait performance of individuals with chronic stroke in terms of temporal-spatial parameters and segmental angles. This training strategy might be adopted as a safe, specific and promising strategy for gait rehabilitation after stroke. It is important to mention that the adopted training protocol kept the interest and motivation of the individuals in this study throughout all of training period.

## Competing interests

The authors declare that they have no competing interests.

## Authors' contributions

COS was responsible for conception and design of the study, gait training, acquisition of data, analysis and interpretation of data, and drafting the article. CLPM was responsible for gait training, acquisition of data, analysis and interpretation of data, drafting the article. TFS and JAB were responsible for interpretation of data and revising it critically for scientific method and content. AMFB were responsible for conception and design of the study, acquisition of data, analysis and interpretation of data, and drafting the article. All authors read and approved the final manuscript.

## Acknowledgements

This work was supported by CNPq (Process #470421/2006-1). C.O Sousa and A.M.F. Barela are grateful to CNPq for their Master scholarship (130483/2008-7) and Post-Doc fellowship (151893/2006-2), respectively, and C.L.P. Medeiros is grateful to FAPESP for her doctoral scholarship (200704503-6). All authors acknowledge P.H. Lobo da Costa for making the use of the laboratory where this study took place possible.
